# Challenges to and advancements of the official veterinary service of the Federal District, Brazil

**DOI:** 10.29374/2527-2179.bjvm003221

**Published:** 2021-12-15

**Authors:** Cristyanne Barbosa Taques, Luiz Cláudio Coelho, Hélio Vilela Barbosa-Júnior, Marcos Eielson Pinheiro de Sá, Márcio Botelho de Castro, Cristiano Barros de Melo

**Affiliations:** 1 Veterinarian, Msc., Secretaria de Estado da Agricultura, Abastecimento e Desenvolvimento Rural do Distrito Federal (Seagri/DF), Brasília, DF, Brazil.; 2 Veterinarian, MSc., Ministério da Agricultura, Pecuária e Abastecimento (MAPA), Brasília, DF, Brazil.; 3 Veterinarian, DSc., MAPA, Esplanada dos Ministérios, Brasília, DF, Brazil.; 4 Veterinarian, DSc., Programa de Pós-graduação em Ciências Animais (PPGCA), Universidade de Brasília (UnB). Brasília, DF, Brazil.

**Keywords:** assessment, quality improvement, veterinary service, avaliação, melhoria da qualidade, serviço veterinário

## Abstract

Animal diseases can be both a precursor to social instability and a result of social instability. For disease outbreaks to be prevented or even quickly controlled, an efficient and well-structured veterinary service is fundamental. The monitoring of the efficiency of an official veterinary service (OVS) by official audits is a *sine qua non* condition for the progress of an efficient way to control and eradicate diseases. In this sense, the present work aims to study the advances and challenges of the OVS of the Federal District (DF), Brazil, from a study of the scores obtained by the evaluation of the Programme of Quality Evaluation and Improvement of Official Veterinary Services which is based on the World Organisation for Animal Health - Performance of Veterinary Services Pathway (Quali-SV/OIE-PVS), which was used to officially assess the quality and improvement of the OVS of the DF. In Brazil, the official evaluation was conducted by the Coordination of Evaluation and Improvement of Veterinary Services, Ministry of Agriculture, Livestock and Food Supply, considering manpower, infrastructure and financial resources. Five possible scoring levels were audited: authority, technical and operational capacity, interaction with stakeholders and access to markets. These components were described and assessed as critical competencies in the OVS assessment. Strengths and weaknesses observed during the audit were listed, and levels of OVS progress were rated and assessed. Despite the predominance of strengths in the OVS audit, the weaknesses detected require urgent corrective action, especially with regard to the autonomy of the OVS.

## Resumo

As doenças animais podem ser simultaneamente precursoras de instabilidade social e resultado da instabilidade social. Para que surtos de doenças sejam prevenidos ou mesmo rapidamente contingenciados, um serviço veterinário eficiente e bem estruturado é fundamental. O acompanhamento da eficiência de um Serviço Veterinário Oficial por auditorias oficiais é condição sine-qua-non para o avanço de forma eficiente para controle e erradicação de doenças. Nesse sentido, o presente trabalho objetiva estudar os avanços e os desafios do Serviço Veterinário Oficial do Distrito Federal (DF), a partir do estudo das pontuações obtidas pela avaliação Quali-SV/OIE-PVS, a qual foi utilizada para avaliar oficialmente a qualidade e a melhoria do Serviço Veterinário Oficial (SVO) do DF. A avaliação oficial foi conduzida pela Coordenação de Avaliação e Melhoria dos Serviços Veterinários, Ministério da Agricultura, Pecuária e Abastecimento (MAPA), considerando os recursos humanos, infra-estrutura e financeiros. Foram auditados principalmente cinco níveis de pontuação possíveis, sendo eles para autoridade, capacidade técnica e operacional, interação com as partes interessadas e acesso aos mercados. Estes componentes são descritos e avaliados como competências críticas na avaliação SVO. Foram listados os pontos fortes e fracos observados durante a auditoria, e os níveis de progresso do SVO foram classificados e avaliados. Apesar da predominância de pontos fortes na auditoria do SVO, os pontos fracos detectados requerem medidas corretivas urgentes, especialmente no que diz respeito à autonomia do SVO.

## Introduction

Animal diseases can be both a precursor to social instability and a result of social instability. Veterinary services and animal health programmes can be disrupted as a result of armed combat, civil disorder and failed governance. When health/veterinary services fail, the occurrence of endemic disease and the risk of disease entry from a border are likely to increase (Lubroth et al., 2017).

Hergot et al. (2021) studied the actions of the official veterinary service (OVS) to mitigate outbreaks of infectious laryngotracheitis (ITL - GaHV-1) and improve biosecurity on laying hen farms in the Minas Gerais State (Brazil) and observed that the actions of the OVS were fundamental to preventing the spread of the virus to other regions. The main actions were: vaccination of chickens quarantined on the farms; manure treatment and controlled transportation of manure, chickens and other waste from farms; sanitary education programmes; farms outside the quarantined area were kept infection free for eight years (2010-2018); restrictive measures to improve biosecurity and monitoring of ILT cases. The OVS and defence actions helped prevent the spread of GaHV-1 to other areas and thus, avoided greater economic, health and social damage. In 2014, the World Organisation for Animal Health (OIE) evaluated the Brazilian OVS using the Performance of Veterinary Services (PVS) methodology, reporting that its technical capacity was of good quality.

Veterinary services are responsible for safeguarding animal resources and play a vital role in humanity’s safety and economic and social well-being. Unstructured and unfunded official veterinary services (OVS) decrease confidence in sanitary guarantees, resulting in an inability to respond to emergencies and uncontrolled epidemics in herds, and significant trade losses. Animal diseases cause significant and unpredictable negative impacts on national economies and may lead to social and political instability. Animal health requires appropriate attention and investment (Word Organisation for Animal Health, 2016a).

The World Organisation for Animal Health (OIE) has reaffirmed its commitment to protecting animal health and welfare, economic prosperity, and social and environmental well-being of human populations worldwide. The OIE develops international standards and recommendations for countries based on the latest scientific advances in collaboration with reference centres and partners (Word Organisation for Animal Health, 2016b).

The OVS must comply with principles to ensure the quality of their activities and based on these principles and standards, in 2007, the OIE developed a leading training platform for sustainable improvement of national veterinary services, named the Performance of Veterinary Services (PVS) Pathway (OIE-PVS). The Brazilian OVS comprises the Ministry of Agriculture, Livestock and Food Supply (MAPA) and state agencies for agricultural health and accredited veterinarians. The OVS’s mission is to ensure animal products’ safety for consumers and access to domestic and foreign markets through the prevention, control and eradication of animal diseases, in addition to regulation of the use of inputs and activities that may affect animal health and welfare. In 2017, MAPA implemented the Programme of Quality Evaluation and Improvement of Official Veterinary Services (Quali-SV) of the federative instances of the Agricultural Health Care System (Suasa). The Quali-SV programme aims to monitor the quality indicators of the official veterinary services for herd health control and/or eradication.

Audits to evaluate the quality of OVSs have been implemented and conducted every three years in Brazil by MAPA with an adaption of the OIE/PVS tool used in other OVSs of OIE member countries. The evaluations involve human, physical and financial resources, besides the OVS’s technical and operational capacity. Following the audits, the OVSs must implement corrective measures based on recommendations and service improvement (Brasil, 2017a; Brasil, 2017b).

The Federal District (DF) is one of the smallest federative units of Brazil with a well established OVS. We conducted a critical study of the strong and weak points detected in the Quali-SV assessment record of the OVS/DF conducted by the Coordination of Evaluation and Improvement of Veterinary Services (CASV/MAPA).

## The evaluation by Quali-SV/OIE-PVS

In the DF, the Undersecretary of Agricultural Sanitary Defence (SEAGRI/SDA/DF) coordinates and supervises agricultural defence. Documentary and bibliographic research and data descriptive analysis related to the MAPA audit in the OVS/DF were conducted on websites of organisations such as the World Trade Organization (WTO), Codex Alimentarius, OIE, MAPA and SEAGRI/SDA/DF. Secondary sources such as books, journals and documents specialising in the topic were also consulted.

The OVS/DF audit was conducted according to MAPA technical criteria supported by the OIE. The methodology included report and information evaluations, interviews and document checks during the audit guided by a standardised questionnaire and sampling inspections at locations previously defined by CASV/MAPA in cooperation with the DF Superintendence of Agriculture (MAPA/SFA/DF).

Quali-SV was used to assess the quality and improvement of OVS, based on the methodology proposed by CASV/MAPA to evaluate the unified system of attention to agricultural health and general animal health guidelines. Quali-SV comprises four fundamental components: 1. Human, infrastructure and financial resources; 2. Authority, technical and operational capacity; 3. Interaction with stakeholders; and 4. Health certification. The evaluation considered advancement levels assessed in each item, issuing grades of quality ranging from one to five. Strong and weak points were listed and classified.

A technical audit of compliance with the Department of Animal Health (DSA) determinations, Secretary of Agricultural Defence CASV/MAPA, was conducted from December 10-14, 2018, in the OVS-DF. Central, intermediary and local instances of human, physical and financial resources (structure, operating conditions, organisation and others), technical and operational capacity, interaction with stakeholders, ability to meet animal health national and international standards, national programmes for prevention and disease control and eradication measures implemented in the country were evaluated. The last OVS/DF audit conducted on March 12-14, 2013, subsidised the 2018 assessment (Secretaria de Estado da Agricultura, Abastecimento e Desenvolvimento Rural, 2018a).

## Challenges to and advancements of the OVS of the Federal District

The OVS/DF is structured based on state legislation of the official programmes of animal disease control, and rules and manuals that deal with their execution. The OIE-PVS tool audit data are presented in Table 1. Computerised systems improved several functions including execution, organisation, management, registration processes, transparency, certifications, herd movement control and sanitary events and were observed in all audited units. However, inconsistent access to the internal network was detected in some units.

**Table 1:** QUALI-SV/OIE-PVS auditing data: official veterinary services (OVS), Federal District, Brazil, 2018.

COMPONENTS	COMPETENCIES	RATED ITEM	AUDIT NOTE
1. Human, infrastructure and financial resources	1.1Human resources	1.1.1 Quantitative and distribution	3
		1.1.2 Stability of structures and sustainability of health policies	4
		1.1.3 Technical and continuous training	4
		1.1.4 Skills and technical independence	4
	1.2 Infrastructure	1.2.1 Infrastructure	4
		1.2.2 Transportation	4
		1.2.3 Equipment and access to communication	4
		1.2.4 Information systems	3
	1.3 Financial resources	1.3.1 Investment resources	2
		1.3.2 Funding resources	3
		1.3.3 Animal health funds	2
2. Authority, operational technical capacity	2.1 Organizational structure	2.1.1 Organizational structure and internal coordination capacity	3
	2.1 Authority and quality management	2.2.1 Legal basis, regulation, application of legislation, manuals and standard operating procedures	3
		2.2.2. Organization of processes and units	3
		2.2.3. Supervision and internal Control	4
	2.3. Technical and operational capacity	2.3.1. Laboratory diagnosis and sample shipment	4
		2.3.2. Foreign exchange and border control	3
		2.3.3. Biological products for veterinary use (vaccines, antigens and allergens)	4
		2.3.4. Registration control of farmers, farms and animals	4
		2.3.5. Transit control of animals (terrestrial and aquatic) and products of animal origin, identification and traceability	3
		2.3.6. Control of events and agglomerations	4
		2.3.7. Planning and directing veterinary surveillance	4
		2.3.8. Capacity for early diseases detection and immediate notification	4
		2.3.9. Ability to respond to diseases and emergencies	4
	2.4. Prevention, Control and diseases eradication	2.4.1. Animal Health and Epidemiology Information System (structure, organization and functioning)	4
		2.4.2. PNCEBT	3
		2.4.3. PNCRH	4
		2.4.4. PNEEB	4
		2.4.5. PNEFA	3
		2.4.6. PNSA	3
		2.4.7. PNSCO	2
		2.4.8. PNSE	4
		2.4.9. PNSS	4
		2.4.10. PNCMB	N/A
		2.4.11. Aquaculture with Health	1
		2.4.12. PNSAp	1
3. Interaction with stakeholders	3.1. Interaction with the community	3.1.1. Health education and media (dissemination and advertising)	3
		3.1.2. Participation with the community and stakeholders consultation	3
		3.1.3. Participation and consultation with institutions and representations	3
	3.2. Interaction with Veterinarians	3.2.1. Qualification and registration of veterinarians	3
	3.3. Interaction with institutions	3.3.1. Inspection system (food security)	3
		3.3.2. Unified Health System (zoonosis, health surveillance, and others)	3
4. Markets access	4.1. Capacity of certification for markets access	4.1.1. Certification capacity	4

Legend: PNCEBT = National Programme for Control and Eradication of Brucellosis and Animal Tuberculosis; PNCRH = National Programme for Herbivore Rabies Control; PNEEB = National Programme for Prevention and Surveillance of BSE; PNEFA = National Foot and Mouth Disease Eradication Programme; PNSA = National Poultry Health Programme; PNSCO = National Goat and Sheep Health Programme; PNSE = National Equine Health Programme; PNSS = National Pig Health Programme; PNCMB = National Programme for Hygienic Sanitary Control of Bivalve Molluscs; PNSAp = National Apiculture Health Programme; N/A = Not assessed.

The Federal District has a financial compensation fund for farmers with brucellosis and tuberculosis in their herds, but the fund would possibly not cope with a great animal health emergency, where it is usually necessary to sacrifice a large number of animals to control such outbreaks. Concerning human resources, a short and medium-term deficiency in the number of technical, support and administrative employees, no replacement of retiring employees and deficient public tender planning were considered major obstacles to meeting the work demand. Most of the Agricultural Health and Inspection Directorate (Disaf) workforce had been selected and employed in public tenders. A lack of Disaf’s operation and expansion planning, and distribution analysis of employees and future retirements were not foreseen to avoid discontinued performance of work activities. Therefore, Disaf’s internal planning was recommended to regulate the organisation chart, technical and administrative areas. Furthermore, the OVS/DF has an established workflow and defined hierarchical command chain based on the technicians’ compact structure and individual effort. However, in the case of work structure expansion or new employees incorporations, there were risks of operation system rupture due to the lack of an organogram and consistent work structure.

The OVS’s audit plan evaluated the expected number of units and documents of Disaf’s activities dynamics. A total of 50 intervention and corrective points were recommended, besides implementing and adopting a State Action Plan (Secretaria de Estado da Agricultura, Abastecimento e Desenvolvimento Rural, 2018b).

The OIE-PVS methodology provides a governance diagnosis and legitimises funding requests in the evaluated country and to international funding organisations. The OIE-PVS is an essential guide to countries with investment requests. A positive evaluation using OIE-PVS tools is a facilitator in import/export trade relations, especially for animal protein, strengthening audited OVS and guaranteeing animal products’ health quality. The OIE country’s evaluation is optional and through documentary analysis and requested by the country. Audited countries in the Americas are action executors of agricultural and livestock defence (Word Organisation for Animal Health, 2019).

In Brazil, the audited government agency is the Ministry of Agriculture, Livestock and Food Supply (MAPA), and defence action execution is conducted in the states by the OVSs. The Programme of Quality Evaluation and Improvement of Official Veterinary Services (Quali-SV) methodology was created considering the same guidelines as the OIE-PVS. Considering the effects of the different critical competencies foreseen in the OIE-PVS tool, for example, a minimum level of evaluation was defined as indicated in the Brazilian Foot-and-Mouth Disease (FMD) Eradication and Prevention Programme, considering a transition from any area to a free zone without vaccination status. The OIE evaluation aims to adapt the OVS’s structural and technical conditions and reduce the vulnerabilities associated with them before reaching the new sanitary condition. The FMD Eradication and Prevention Programme is not restricted to transition areas and adopts the same methodology to reduce the vulnerabilities that can threaten the health status of Brazilian herds (Brasil, 2017c).

The OVS/DF audit showed governance results below the adequate level. Proper management is supported by efficient and adequate control structures, which is also an administered right (Marques Neto, 2010). Governance sets criteria for goal identification and necessary procedures to achieve them (Peters, 2013). The low evaluation of critical competencies compromises the whole OVS’s performance. Mismanagement in the employees hiring and budget allocations, replacement of goods tied by agreement resources and lack of productive class interrelationships were also detected.

Regarding OVS/DF human, infrastructure and financial resources, a score of four was predominant and considered adequate. However, financial resources scored two and three, showing a reduced financial investment contribution. The animal health fund was a weak point detected. Despite provision forms being established for contingency and indemnity funds, resources were limited and insufficient. A reduced interrelationship between the OVS-DF and producing classes, farmers, agro-industries and animal origin transporters’ products was also observed.

Partnerships, networks and alliance construction are related to an institution’s sustainability, favouring the raising of financial and material resources. Therefore, the OVS must demonstrate public health relevancy to society. The creation of an organisation that discusses, suggests and contributes to animal health issues, with the effective participation of representatives of the DF’s productive chains is a path that can be followed by the OVS. A necessary approximation and integration with the productive sector and the alignment and integration of private initiative professionals in animal health defence are required in a unique health context (Drucker, 2002; Instituto Interamericano de Cooperação para a Agricultura, 2008).

It is important to highlight the example of the Brazilian programme’s success for African swine fever eradication, a reflection of government efficiency and agility associated with the pig production chain’s intense participation, and the private sector veterinarians (Moura et al., 2010).

Improvement in the management of the animal health fund supports severe financial and economic losses in events of vesicular disease introduction in the beef cattle-producing region as a consequence of trade embargoes, suspension of animal origin products’ marketing and obligatory sanitary slaughter provided by law (Garcia et al., 2015). Although the producer is refunded, financial reimbursement does not cover high zootechnical value animals.

The OVS-DF capacity and technical authority components were classified as regular to satisfactory (no score below 3). However, a weakness pointed out in the report was the absence of continuous training programmes for OVS technicians, compromising flexibility and effectiveness in meeting animal health defence actions. Capacity and technical authority actions require decision-making for effectiveness, professionals with skills and abilities to assess, systematise and decide the most appropriate behaviours, based on scientific evidence (Castilho, 2015).

In the prevention, control and eradication of diseases, health programmes were heterogeneous, with some satisfactory programmes receiving a score of four, while the OVS-DF had not even implemented others. The animal products’ innocuity is consolidated as a fundamental trade relations requirement, which is guaranteed through the surveillance and inspection of productive systems (Garcia et al., 2015). The OVS’s compliance is configured by actions executed under regulations and legal norms to mitigate risks, and in this case, related to agricultural surveillance procedures (Manzi, 2008). Concepts of compliance include implementation, monitoring, identification, measurement, prioritisation and risk mitigation, generating risk management processes.

Food safety agreements and animal and plant health standards, such as the Sanitary and Phytosanitary Measures (SPS Agreement), establish fundamental trade relations between countries. Such agreements apply standards to protect human, animal and plant health, safeguarding food safety. Additionally, these rules use various standards and methods to inspect products, providing equitable, fair and scientific subsidies, and avoiding the disguise of protectionism (World Trade Organisation. Resources, 2020).

Risk management is only possible through an adequate analysis based on an interdisciplinary scientific approach to identify and quantify relationships between risks and damages to support mitigation alternatives. The Food and Agriculture Organization uses the Risk Analysis Programme (RA), a formal method developed to identify hazards and widely used and disseminated to evaluate and measure risks inherent in agricultural defence actions by OVSs (Food and Agriculture Organization of the United Nations, 2015). The application of the RA by the OVS in commercial import/export relations evaluates and mitigates risks to animal and human populations related to animal movement and animal-derived products (Santos et al., 2014).

New technologies, computerised systems and analysis of diseases’ spatial distribution improve agricultural defence actions. Transit routes and monitoring of animal movements are fundamental to subsidising the decision-making process and implementing measures to prevent and control diseases (Carvalho et al., 2012a; Sá & de Melo, 2016). The OIE-PVS tool is essential to maintaining animal protein exports to FMD-free European Union and other countries, improving the computerised control of herd movements in Brazil.

A computerised system for the control of registration and animal movement was identified in the OVS-DF’s units. However, the herd access data and history were not analysed, resulting in a weak interaction and information checking sent to the Platform for Agricultural Management (PGA). The lack of interaction between the OVS-DF and PGA compromised the reliability and information access agility by the productive chains. A PGA pilot project implementation in the OVS-DF would be a technically feasible and economically interesting solution due to geographical proximity with MAPA.

Considering the diversity of available technologies and the DF’s small geographic extension, “Google Earth” is quite viable for remote animal sensing and locating livestock farms (Carvalho et al., 2012b). Developing systems or improving existing technologies through university partnerships and private sectors and implementing analytical intelligence use are possible solutions to connect to the PGA properly.

The OVS-DF had regular scores for interaction with stakeholders, highlighting an improvement need for partners’ expectations. The development of strategies that contribute to fundraising and communication with society are also desirable (Freeman, 2010). Thus, partnerships with institutions representing the agricultural sector are viable solutions, incorporating private initiatives in agricultural defence actions, modernising the whole defence system.

The market access component was one of the strongest points of the OVS-DF in the audit. Procedures were adequately executed and several products were satisfactorily certified. Considering the vigorous participation and expressive market conquests of Brazilian agribusiness in international trade in recent years, the guarantee of animal origin product safety through surveillance and inspection actions in production systems is a fundamental requirement in the consolidation of trade relations (Eidt et al., 2015).

The elaboration of multi-year plans that contemplate macro aspects of public policies, strategies, guidelines, relevance, resources and responsibility delimitation is one way to improve governance. Such policies must include the “One Health” approach considering 75% of human infectious disease agents have an animal origin; five new diseases in humans appear annually and most have an animal origin; and 80% of zoonotic disease agents can be used as biological weapons (Organização Mundial da Saúde, 2018).

Weapons and biological wars generate devastating economic, political and socio-psychological repercussions. On this basis, elaborating plans that consider risk, including human resources training, investments in bioterrorism material identification and security equipment, is essential (Rambauske et al., 2014).

The political dependence of SEAGRI/SDA/DF compromises the autonomy of the OVS-DF, a fundamental competence in inspection actions’ good practices. Human resource planning deficiencies and no management under the Undersecretary of Defence’s control should be highlighted in the OVS-DF as hindering service demands. Despite a public contest in 2009 and calls for an OVS-DF technician until 2013, an OIE-PVS/Quali-SV audit detected a human resource insufficiency in the OVS-DF (Secretaria de Estado da Agricultura, Abastecimento e Desenvolvimento Rural, 2018b).

The resolution of non-conformities listed in the OVS-DF’s audit is necessary and urgent. Animal health is a guarantee required for local, regional and international market access. Governance is the fundamental stone of all administrative, budgetary and technical actions. Considering the opportunity, analysis of different solution possibilities for the OVS/DF suggests creating an agricultural defence agency with administrative, technical and financial autonomy to maintain the requirements for quality and animal products, herds and plantations’ health. The existence of agricultural defence agencies or institutes in most of the federation units corroborated this proposition. Actions of agricultural defence through agencies or institutes are executed in 22 of the 27 federal states in Brazil.

Administrative autonomy is essential in agricultural defence actions and is hugely strengthened in these Brazilian federal units. As a result, a quality gain in production will be established in the OVS-DF and Brazil. As an example of OVS excellence, Santa Catarina State was recognised, by the OIE in 2007, as an FMD-free zone without animal vaccination, being consolidated as a significant producer and animal protein exporter (Companhia Integrada de Desenvolvimento Agrícola de Santa Catarina, 2020).

In 2016, the excellent performance of Paraná State’s OVS resulted in OIE recognition as a classical swine fever-free Federative Unit, becoming part of a block of entirely disease-free zones besides Santa Catarina and Rio Grande do Sul States. These states are also fulfilling the requirements to be declared OIE FMD-free zones without vaccination in accordance with sanitary rules and controls detected in the states OVSs audited by MAPA (Agência de Defesa Agropecuária do Paraná, 2019).

The adequate power and support of the official service are *sine qua non* conditions for the development and protection of the regions and countries. When this is not observed, the results can be catastrophic. In Nigeria, terrorist groups affected the country’s ability to control disease, and hampered the ability of public services to function. The lack of full operational government services contributed to the reintroduction of H5N1 into poultry areas in 2014. In another case, in Syria, deficiencies in veterinary services following the 2014 uprising meant that they were no longer able to conduct brucellosis vaccination in herds and flocks, and probably was the cause of a reported human brucellosis increase (Lubroth et al., 2017).

Given the severe repercussions that certain pathogens can have on public and animal health, as well as on the food economy, it is essential to make proper preparations by drawing up emergency plans and testing them using simulation. It is important to have high quality veterinary services which comply with OIE standards as a basis for surveillance and early detection of any pathogen, to limit risks and ensure the best response to any threats, whatever their origin (Vallat & Chaisemartin, 2017).

Brazil, in a broader way, considering the OVS, has been investing in training, data analysis and staff training with a scientific basis, including themes related to the inspection of products of animal origin (Rodrigues et al., 2017, 2018, 2021), international agricultural surveillance (Melo et al., 2014a, 2014b, 2015; Sá et al., 2018), agricultural defence (Moraes et al., 2017; Oliveira et al., 2021) and diagnosis and official laboratory structure (Dias et al., 2014; Oliveira et al., 2018, 2021). This shows that the country has been preparing for the new sanitary challenges in livestock and in this sense, the DF continues to adapt to international requirements, as demonstrated in this paper, especially considering the stage Brazil is at in the National Programme for the Eradication of FMD, in which the intention is to gradually remove vaccination against FMD from cattle and buffalo herds in Brazilian states, in accordance with the proposal approved by the OIE (Brasil, 2019).

This work exemplified the application of OIE-PVS tools and Quali-SV to audit the quality, strengths, weaknesses, objectives and proposals of the OVS-DF. In addition to OIE-PVS tools, Quali-SV is a standardised and transparent technical tool, inducing process improvement in animal health defence. Quali-SV is also an agricultural and livestock defence validation instrument, including a decision-making tool to manage programmes and legal aspects.

OVS-DF showed more strong points in the evaluated competencies than weaknesses. Improvements and the application of corrective measures are needed, propitiating the OVS-DF evolution. The approximation and partnerships between the OVS-DF and the private sector will provide better conditions for animal sanitary defence actions. This measure will guarantee safety levels, sanitary condition improvement and capacity for a crisis scenario, using intelligence to control animal movements, animal health and animal origin product quality besides the inspection and monitoring actions.
